# A human relevant in vitro alveolar epithelial barrier model to assess inhaled pollutant hazard

**DOI:** 10.1038/s41598-025-23768-4

**Published:** 2025-11-14

**Authors:** Joshua W. P. Bateman, Kirsty Meldrum, Sarah M. Mitchell, Ulla Vogel, Martin J. D. Clift

**Affiliations:** 1 In Vitro Toxicology Group, Institute of Life Science 1, Swansea University Medical School, Swansea, SA2 8PP UK; 2 The National Research Centre for the Working Environment, Lersø Parkalle 105, Copenhagen Ø, DK-2100 Denmark

**Keywords:** Multi-cellular model, Alveoli, In vitro, Toxicology, Respiratory system models, Respiratory tract diseases, Inflammation

## Abstract

Relevant in vitro models could reduce the requirement for in vivo testing and allow higher throughput for imperative toxicological or pharmaceutical hazard testing. Models of the alveolar barrier are invaluable when assessing the toxicity of inhaled xenobiotics, though there is a requirement that these models are well-characterised and accurately resemble the relevant human cellular architecture. Here, a triple cell co-culture has been developed using hAELVi, NCI-H441 and differentiated THP-1 cells as models of type 1 and type 2 pneumocytes, and alveolar macrophages, respectively. Through pre-staining each cell type, confocal microscopy was first used to determine cell seeding ratios to hAELVi and NCI-H441 required to achieve a human-relevant 16.44:1 ± 3.29 ratio at the time of air-liquid interface exposure. CellTrackers were then used to ensure that the density of differentiated THP-1 cells was in line with previously published anatomical research at 1 cell/18 × 10^3^ µm^2^. We were able to show that the triple culture forms a tight barrier and that the macrophages can respond to a (pro)-inflammatory stimulus (lipopolysaccharide). Given the anatomical relevancy and its ability to react to stimuli, this model may provide a useful platform to assess the toxicological hazard potential of a range of inhaled, respirable xenobiotics.

**Supplementary Information:**

The online version contains supplementary material available at 10.1038/s41598-025-23768-4.

## Introduction

 The lower lung, specifically, the alveolar epithelial barrier, is a primary location of the body exposed to air pollutants, and is often the site of toxicity for particulate matter (PM) below 2.5 μm in aerodynamic diameter (PM_2.5_)^1,2^. Historically, in vivo approaches, especially those involving the use of rodent models, have been a crucial methodology in assessing the inhalation risk of air pollutants. However, the use of such models does come with drawbacks. Firstly, the ethical consideration of in vivo studies must be acknowledged, and as such, their use must be justified. Within the EU, for example, cosmetic testing on animals has been banned since 2004^3^. Other groups, such as the National Centre for the Replacement, Refinement and Reduction of Animals in Research (NC3Rs), aim to improve the ethical framework surrounding in vivo studies, with explicit emphasis on the replacement, reduction, and refinement of animal testing. Secondly, there are significant differences between the lungs of a human compared to the lungs of a rodent. For example, human lungs possess 23 branching generations, whereas mouse lungs possess 16, altering the deposited fraction of PM at the alveolar level. Further, the histological composition of a rodent lung differs in terms of its cell ratios and cell types compared to a human lung; human airways are vertical, compared to the horizontal airway of a rodent, and ventilation dynamics highly contrast^4,5^. These differences have been reviewed in high-depth by Stucki et al.^6^. These anatomical and physiological differences could result in different responses to inhaled pollutants.

In line with the ‘replacement’ strand of the 3R principles, in vitro models pose as appropriate systems to be implemented within air pollution exposure studies, providing data that can be extrapolated to real-life human exposures^7^. To allow this, these in vitro models should accurately represent the tissue which they are intended to model. This can be achieved firstly with co-culture models, compared to monoculture models. Co-culture models allow numerous cell types to be cultured within the same system, resembling the cellular components that would be present within an in vivo system. Cells pertinent to the alveolar epithelial barrier include alveolar type 1 cells (ATI), alveolar type 2 cells (ATII) and alveolar macrophages (AMs). Squamous ATI occupies approximately 90–95% of the alveolar surface and facilitates gas movement between the alveoli’s lumen and the blood. This is made optimal through the extremely thin nature of ATI at 0.2 μm^8^. Cuboidal ATII vary vastly in function and are easily identified through electron microscopy by numerous lamellar bodies, which store surfactant proteins that are secreted into the alveolar epithelium^9^. Lung surfactant is needed to reduce alveolar surface tension, preventing alveolar collapse and maintaining normal lung function^9^. AMs are the resident innate alveolar phagocytic immune cell, acting as the first line of cellular defence against inhaled xenobiotics by playing a role in the modulation of inflammatory processes, as well as tissue repair. AMs possess receptors that are able to recognise damage-associated molecular patterns, pathogen-associated molecular patterns, cytokines and chemokines allowing them to mount pro/anti-inflammatory or pro/anti-fibrotic responses depending on the nature of the stimulus^10^. Within the context of air pollution, AMs can indeed induce a (pro)-inflammatory response to air pollution exposure (PM, gases and pathogens) in vitro, as well as contribute to detoxification through endocytic uptake^11,12^.

The Guidance Document on Good In Vitro Method Practices (GIVIMP) has specific guidance for cultures that are used at the air-liquid interface (ALI), i.e., lung, eye and skin models, as well as exposure methods specific to inhalation toxicology^13^, and will therefore be adhered to in this work. By combining in culture ATI, ATII and AMs together, a relevant model for the toxicological assessment of inhaled pollutants could therefore be formulated. Further, culturing these cells at the ALI has been shown to push airway cells towards a phenotype more applicable to in vivo conditions compared to submerged lung cultures^14^. Though well-characterised, commercial alveolar ALI cultures are available, e.g. AlveolAir (Epithelix), EpiAlveolar (MatTek), this work intends to produce a cost-effective, reproducible, well-characterised and human relevant alveolar model using immortalised cells.

Here, the development of an alveolar triple cell co-culture is described where hAELVi, NCI-H441 and dTHP-1 cells will be used to model ATI, ATII and macrophages, respectively. Previously, hAELVi and NCI-H441 have been used in co-culture, though, the relevancy of the each cell ratio to human physiology/anatomy were not highlighted^15^. dTHP-1 cells have also been used within alveolar co-cultures previously, modulating the (pro)-inflammatory response and toxicodynamic response^16^. This work aims to characterise and develop an in vitro alveolar triple cell-co-culture that will be subsequently utilised for inhalation toxicological studies. To achieve this, the following objectives will be fulfilled:

1. hAELVi cells will be characterised in terms of their growth, viability, barrier function, morphology and (pro)-inflammatory response in both submerged and ALI conditions.
2. A co-culture of hAELVi and NCI-H441 cells will be characterised to ensure that an anatomically relevant ratio of each cell type is present within the co-culture.
3. An assessment of the number of dTHP-1 within the triple cell co-culture will be undertaken again to ensure relevance to the anatomy of the human lung. The functionality of the culture will then be assessed using a (pro)-inflammatory stimulus.

## Methods

### Chemicals and reagents

Unless otherwise stated, all chemicals and reagents were purchased from Sigma-Aldrich (UK).

### Cell culture

hAELVi cells (passage 6–30) were obtained from InSCREENeX (INS-CL-1015) (Germany) and were cultured in huAEC medium (INS-ME-1013; InSCREENeX) containing 10% huAEC supplements (InSCREENeX) in flasks coated with huAEC Coating Solution (INS-SU-1018; InSCREENeX).

NCI-H441 cells (CRM-HTB-174) (passage 3–30) were obtained from American Type Culture Collection (ATCC) (Manassas, VA) and were cultured in RPMI 1640 medium (A1049-01; Gibco) containing D-glucose, (4.5 g/L), HEPES buffer (2.3 g/L), L-glutamine (1%), sodium bicarbonate (1.5 g/L), sodium pyruvate (110 mg/L) and additionally supplemented with FBS (16140071; Gibco) (10%) and penicillin/streptomycin (15140-122; Gibco) (1%).

THP-1 cells (TIB-202) (passage 3–30) were obtained from ATCC and were culture in RPMI 1640 medium (31870-025; Gibco) supplemented with FBS (10%), L-glutamine (25030-024; Gibco) (1%) and penicillin/streptomycin (1%). THP-1 cells were differentiated into a macrophage-like phenotype (dTHP-1) by culturing in phorbol 12-myristate 13-acetate (PMA) (P8239; Merck) (10 nM) for 48 h, then 24 h in fresh medium to recover without PMA. dTHP-1 cells were then ready to be seeded onto the apical side of the epithelial co-cultures.

Cell cultures were checked daily for growth, contamination and morphology *via* microscopy. All cultures were assessed for mycoplasma contamination routinely using a MycoAlert Mycoplasma Detection Kit (Lonza, Switzerland).

The cell seeding and culture methodology was adapted from Mitchell et al.^17^. Co-cultures were grown on 12-well cell culture inserts (3 μm pore size, 0.9 cm^2^ growth area, PET) (353181; Falcon, UK) coated with huAEC coating solution. Culture inserts contained 1.5 ml cell culture media in their basal compartment, and 0.5 mL of cell suspension in the apical insert all experimentation undertaken.

Monocultures of hAELVi or NCI-H441 cells were seeded at 2.5 × 10^5^ cell/ml (1.39 × 10^5^ cell/cm^2^) in huAEC or RPMI 1640 medium, respectively, and allowed 72 h to adhere. The basal media was then replaced, and cells were taken to the ALI by removing the apical media.

Co-cultures were seeded and cultured in a 50/50 mixture of complete huAEC and RPMI 1640 cell culture media. The co-culture was seeded at a density of 5 × 10^5^ cell/mL (2.78 × 10^5^ cell/cm^2^), though, the ratio of each cell type is characterised and justified within the ‘Deducing Seeding Concentrations of hAELVi and NCI-H441 Co-Cultures’ section. hAELVi/NCI-H441 co-cultures were seeded and allowed 70 h to adhere where the apical media could then be replaced with dTHP-1 suspension and allowed 2 h for dTHP-1 cells to adhere^16^. The basal media was then replenished, and the apical media removed to take the cultures to the ALI.

### CellTracker staining and confocal microscopy

To confirm the surface coverage of each epithelial cell type, hAELVi and NCI-H441 cells were pre-stained using CellTracker Deep Red (C34565) and CellTracker Violet BMQC (C10094) dye (Invitrogen, UK), respectively. Deep Red and Violet CellTrackers were added to each cell suspension at a concentration of 10 µM and 20 µM, respectively. Suspensions were then incubated for 30 min at 37 °C, 5% CO_2_ and gently mixed every 10 min. Suspensions were then centrifuged at 340 RCF before being resuspended in 1 mL fresh cell culture media, centrifuged again at 340 RCF, and resuspended in 1 mL fresh cell culture media. A sample of these pre-stained cells were then counted before dilution to the final seeding concentration and seeded within 12-well cell culture inserts.

After 72 h, the basal media was replaced with fresh 50/50 cell culture media and the cells were taken to the ALI. At 96 h, all media was removed and the inserts washed with PBS three times by adding 1 mL PBS to the basal compartment, and 0.5 mL PBS to the apical insert, taking care not to wash away adhered cells, and then discarding PBS. The inserts were fixed by incubating in 3% paraformaldehyde (158127; Merck) (1 mL in the basal compartment, and 0.5 mL in the apical compartment) for 15 min. The inserts were then again washed three times with PBS and then stored at 4 °C in sterile PBS containing glycine (0.5 M) (G7126; Merck), protected from light. When ready to be imaged, the insert membranes were cut from their plastic scaffold and placed cell side up on a glass slide. A drop of DPX mounting solution was added to the membrane and sealed with a coverslip. DPX was allowed to dry overnight protected from light at room temperature.

The slides were imaged on a Zeiss LSM710. An 8 × 8 tile scan was captured for each seeding ratio biological replicate. From this 8 × 8 tile scan, a 1 × 10^6^ µm^2^ region of interest was defined using Fiji ImageJ software. The blue (hAELVi) and red (NCI-H441) colour channels were then split and converted to black and white images using the ‘Threshold’ tool. Once a black and white image was generated, Fiji’s integrated ‘Analyse particles’ tool was used to quantify the surface area of the black within the image. This gave the surface area covered by either cell type (Fig. 3).

**Fig. 1:**
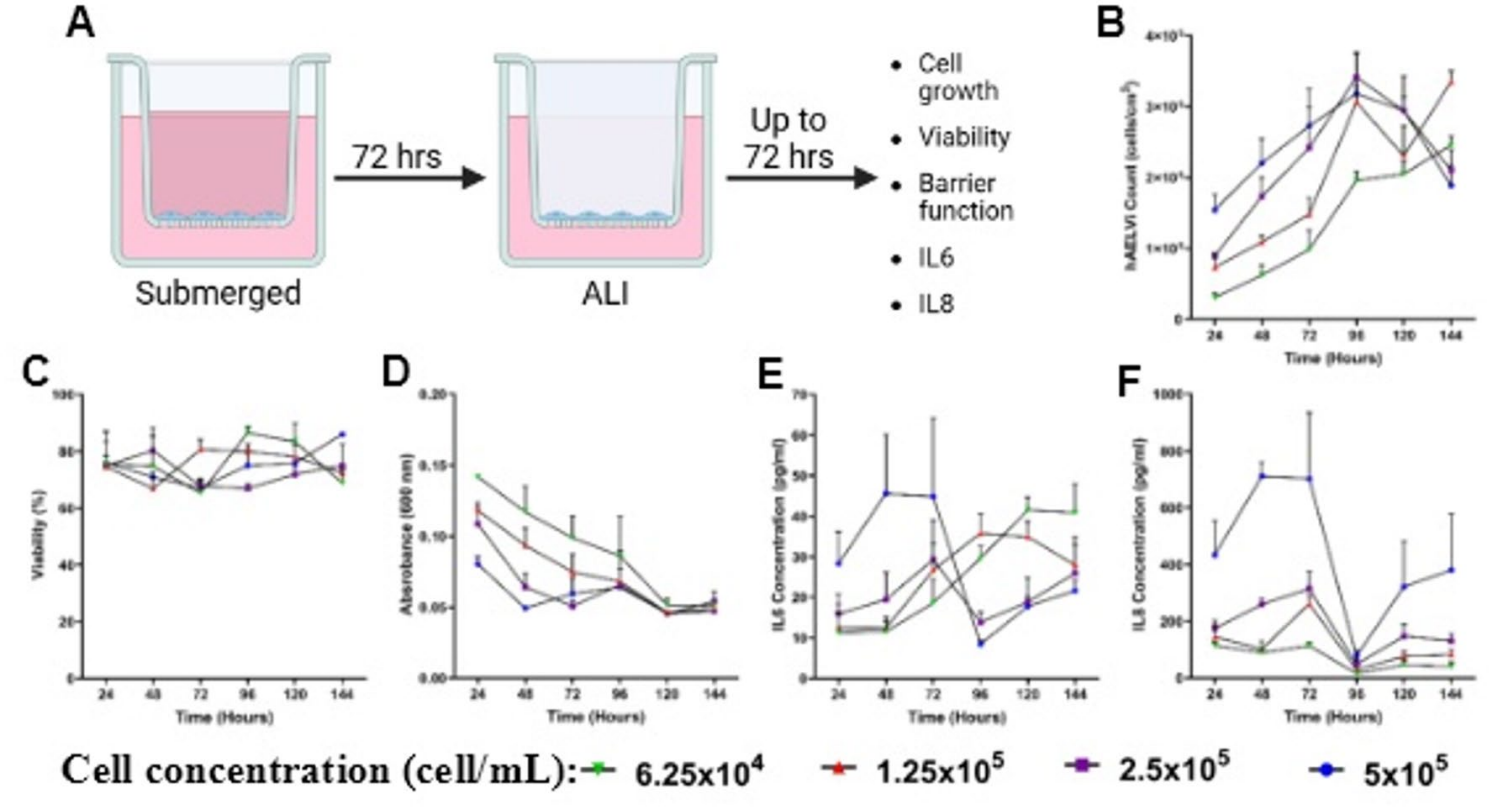
Characterisation of hAELVi cells at the air-liquid interface. (**A**) Schematic of hAELVI culture methods and ALI exposure. Total cell counts (**B**) viability (**C**), barrier integrity (**D**), IL-6 release (**E**) and IL-8 release (**F**) of hAELVi cells every 24 h for a total 144 h growth time. hAELVi cells were grown under sunder submerged conditions for 72 h before being taken to the ALI for the final 72 h (96 to 144 h time points). Basal media was replenished at the 72-hr time point. Data points show the mean of each time point ± SEM over three biological replicates (n = 3). Where error bars cannot be seen, this is due to the SEM bars being smaller than the data icon.

CellTrackers were also used to quantify the number of dTHP-1 cells in the triple cell co-culture after cultures had been taken to the ALI. Prior to the addition of dTHP-1 cells onto the epithelial co-culture, dTHP-1 cells were pre-stained with CellTracker Deep Red using the same protocol as used with the NCI-H441 cells. dTHP-1 cells were then seeded at concentrations of 5000, 6430 and 30,000 dTHP-1/well (within 100 µL 50/50 cell culture media), allowed to adhere for 2 h before apical media was discarded and cultures were taken to ALI. These cultures were incubated for 24 h before wells were fixed and imaged as previously described, though, the number of dTHP-1 was manually counted using the Cell Counter plugin for Fiji to allow a cell number per surface area figure to be calculated.

### Lipopolysaccharide exposure

To assess the capability of the triple cell co-culture to respond to an inflammatory stimulus, cells were exposed to lipopolysaccharide (LPS). LPS (Sigma-Aldrich, L4391) was added directly to the basal cell culture insert CCM to a final concentration of 1 µg/mL (1.5 mL total) and allowed to incubate for 24 h before assessment of (pro)-inflammatory response^18^.

Aerosol LPS exposure, to better simulate inhalation, was conducted using a VITROCELL Cloud 12, whereby 200 µL of 175 µg/mL LPS in water (spiked with 0.009% NaCl) was nebulised onto the apical surface of triple cell co-cultures^19^. This equated to a theoretical LPS deposition of 0.19 µg/cell culture insert based on a deposition efficiency of 83.6%^20^.

### Cell counts and viability

Cellular viability was measured using the trypan blue exclusion assay. Briefly, cells were detached from cell culture inserts using 0.5 mL trypsin-EDTA, pelleted, and resuspended in 0.25 mL fresh CCM. Cell suspension was then mixed 1:1 with trypan blue (11538886; Gibco) and automatically counted using a LUNA II automated cell counter (Logos Biosystems, South Korea).

### (Pro)-inflammatory response

Supernatants were collected from the basal chamber of the cell culture inserts and stored at −20 °C, before assessment for (pro)-inflammatory mediators TNF-α, IL-6 and IL-8 *via* enzyme-linked immunosorbent assay (ELISA) (DY210, DY206 and DY208; R&D Systems, USA). ELISAs were performed following the manufacturer’s protocol.

### Blue dextran barrier integrity assay

To assess barrier integrity of cultures grown in a cell culture insert, all media was removed from the basal and apical chambers of the insert. The basal media was replaced with 1 mL of fresh CCM (37 °C) and 250 µL of blue dextran (2000 kDa) (Y2-9 MJ-17–0360-01; General Elecric) added to the apical chamber. The cultures were then incubated for 2 h before apical blue dextran was removed and discarded. The basal media was then added to a 96 well plate in triplicate (200 µL per well) before absorbance was measured at 600 nm using a plate reader (FLUOstar Omega, BMG Labtech). An increased absorbance would indicate a loss of barrier function shown through increased translocation of blue dextran from the apical to the basal compartment.

### Exposure to carbon black

The triple cell co-culture was exposed to Printex 90 Carbon Black (CB) using a VITROCELL Cloud aerosol exposure system. A stock suspension of CB was made by pre-wetting 8.9 mg CB with 25 µL ethanol (100%). This was made up to 2.6 mL using distilled water making a 3.4 mg/mL suspension. The suspension was sonicated using a Branson SFX 550 Sonifier Kit at 10% power, at intervals of 10 s on, 10 s off for a total sonication time of 4 min.

The stock CB suspension was then diluted to a final concentration for aerosolisation using distilled water. CB suspensions were spiked with 0.009% NaCl to assist nebulisation with deposited concentrations of 390, 780 and 3100 ng/cm^2^ targeted using the integrated quartz crystal microbalance (QCM). Sequential 200 µL nebulisations were performed until the targeted concentration was reached. Cultures were then incubated for 24 h before being assessed for viability, cell count, barrier integrity, and the production of IL-6 and IL-8.

### Statistical analysis

All data are displayed as the mean ± standard error of the mean, unless otherwise stated in the figure legend. All characterisation and endpoints are assessed following three biological replicates, unless otherwise stated. Statistical analyses were performed using GraphPad Prism 10 (Dotmatics) software. Normality of data was assumed. One-way ANOVAs and two-way ANOVAs paired with a Tukeys post hoc test, and t-tests were performed in this work. Figure legends identify the statistical test performed. Results were considered significant if *p ≤* 0.05.

## Results and discussion

### NCI-H441 compatibility with HuAEC coating

Prior to seeding hAELVi cells within cell culture inserts, the inserts were required to be coated with huAEC coating solution to ensure cell adherence. NCI-H441 cells do not require this coating for adherence and growth and have not been characterised in monoculture with this coating. Therefore, it was pertinent to ensure that NCI-H441 cells did not differ in their growth or (pro)-inflammatory response when grown on huAEC-coated cell culture inserts, which would subsequently allow them to be used in co-culture with the hAELVi cells.

**Fig. 2:**
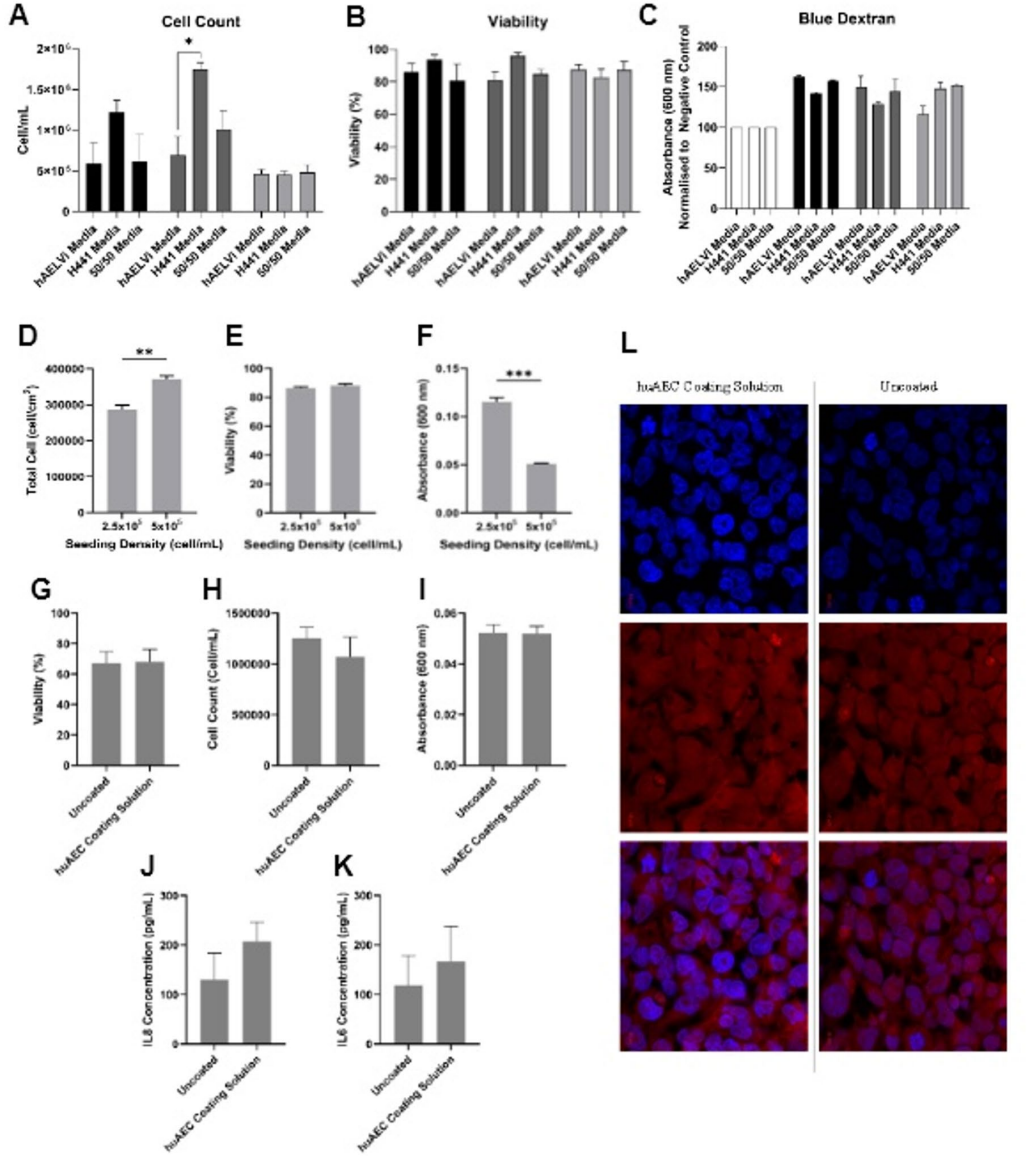
Cell count, viability and barrier function of epithelial co-cultures and monocultures in different media type**s.** Total cell counts (**A**), viability (**B**) and barrier function (**C**) of hAELVi monocultures, NCI-H441 monocultures, or hAELVi/NCI-H441 co-cultures (black, dark grey and light bar bars, respectively) at 96 h post seeding using LUNA II automated cell counter under submerged conditions for 72 h before being exposed to the ALI for 24 h. The barrier integrity (**C**) was measured using blue dextran assay where the absorbances have been normalised to a negative control (cell culture insert containing no cells for each media type) each media type possess varying background absorbances. The cells were cultured in either RPMI 1640 (NCI-H441 media), huAEC media (hAELVI media), or a 50/50 mixture of the two media types (50/50 media). The mean reading of three biological replicates is plotted ± SEM. Difference was assessed for via a two-way ANOVA utilising a Tukeys post hoc test. Seeding density seeded at 2.5 × 10^5^ or 5 × 10^5^ cell/mL (2.5 × 10^5^–2.25 × 10^5^ hAELVi & 2.5 × 10^4^ NCI-H441. 5 × 10^5^–4.5 × 10^5^ hAELVi and 5 × 10^5^ NCI-H441) were compared for total cell count (**D**), viability (**E**) and blue dextran (**F**) for co-cultures seeded at 2.5 × 10^5^ or 5 × 10^5^ cell/mL (2.5 × 10^5^ – 2.25 × 10^5^ hAELVi & 2.5 × 10^4^ NCI-H441. 5 × 10^5^ – 4.5 × 10^5^ hAELVi and 5 × 10^5^ NCI-H441) (submerged conditions for 72 h before being exposed to ALI for 24 h). NCI-H441 monocultures were assessed for their ability to grow on surfaces coated with huAEC coating solution shown through viability (**G**), total cell count (**H**), membrane integrity (**I**), IL-6 release (**J**), IL-8 release (**K**) and DAPI/phalloidin staining (L). * = *p* ≤ 0.05 ** = *p* ≤ 0.01, *** = *p* ≤ 0.001, shown through unpaired t-test. Bars show mean ± SEM across three biological replicates (n = 3).

NCI-H441 cells showed no significant difference (*p* > 0.05) in growth, viability, membrane integrity and (pro)-inflammatory cytokine release (Fig. 2), although, a non-significant increase in both IL-6 and IL-8 release was observed in coated wells. Viability and blue dextran absorbance were similar between the coated and uncoated wells; however, cell counts were not significantly higher in the coated wells compared to the non-coated wells (*p* > 0.05). Morphological analysis of NCI-H441 cells cultured in either uncoated wells or coated wells revealed no changes.

Although there is no description in the literature of NCI-H441 cells being grown on surfaces coated with huAEC coating solution, which contains collagen R (20 µg/mL) and fibronectin (10 µg/mL), hAELVi cells have been found to grow effectively on surfaces coated with collagen I. NCI-H441 cells also grew effectively on the collagen I-treated surface, further indicating compatibility between NCI-H441 and extracellular matrix component-coated surfaces^15^. Work here showed no significant difference in cell growth, viability, membrane permeability, IL-6 and IL-8 release or morphology. Therefore, previous characterisation by Mitchell *et* al.^17^ was used to advise seeding requirements of NCI-H441 cells within the proposed alveolar co-culture model.

### Growth and viability of hAELVi/NCI-H441 Co-culture at the air-liquid interface

Epithelial co-culture seeding density requirements were modelled through extrapolating hAELVi cell and NCI-H441 cell monoculture growth rates at the ALI (Fig. 1, and Mitchell et al.^17^, respectively) (comparative submerged growth rates are shown in supplemental Fig. 1). These calculations gave a theoretical seeding density required to reach a 13:1–16:1 ratio of ATI to ATII^8^. However, the media type required for the optimal co-culture growth had first to be determined. Here, huAEC, RPMI 1640 or a 50/50 mix of the two was utilised.

**Fig. 3:**
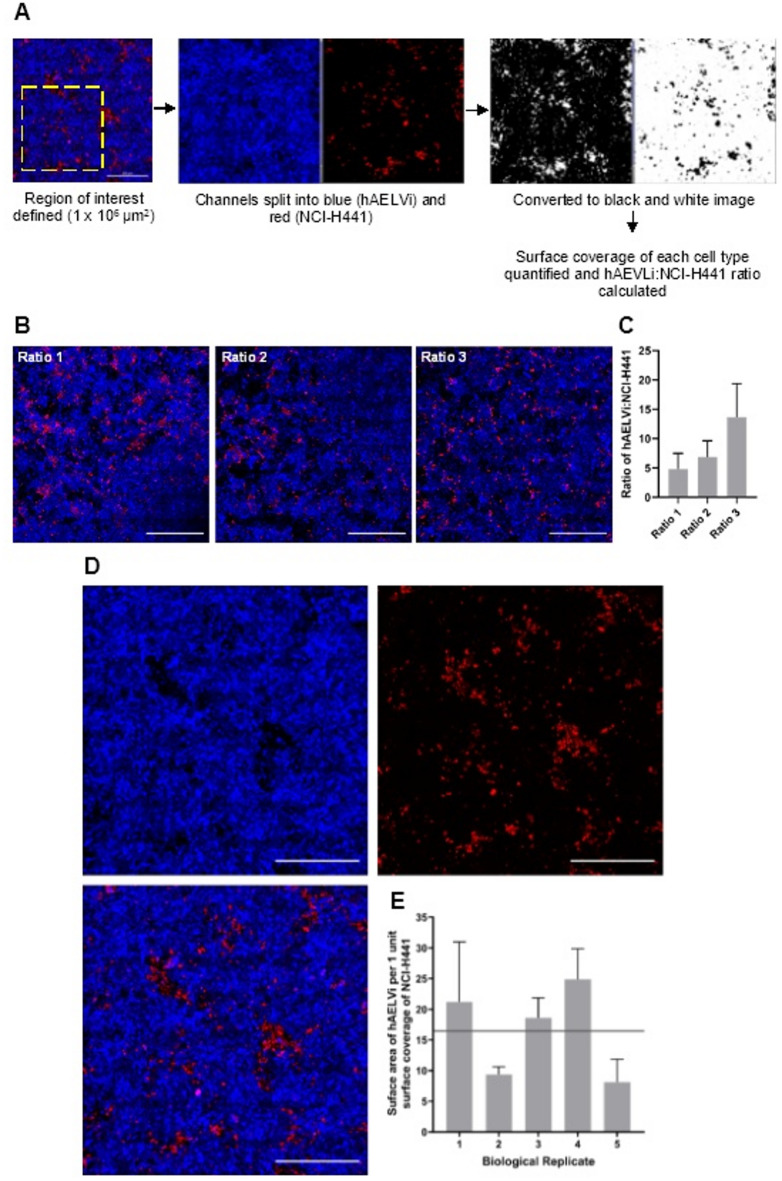
CellTracker staining and cell coverage quantification of epithelial co-culture. (**A**) Scheme to illustrate how each alveolar epithelial cell type were distinguished and quantified. Initially, a tile scan image was assigned a 1 × 10^6^ µm^2^ region of interest, before the blue and red channels were split and converted to black and white images. The black area was then quantified to assess surface coverage of each cell type. (**B**) CellTracker staining of hAELVi (blue) and NCI-H441 (red) co-cultures seeded at ratio 1, ratio 2 and ratio 3 (scale bar = 5000 μm). (**C**) Average ratio of hAELVi to NCI-H441 across three replicates. Each bar shows average across three FOVs ± SEM. Scale bars – 500 μm. (**D**) CellTracker staining of hAELVi (blue) and NCI-H441 (red) co-cultures seeded at 4.5 × 10^5^ and 5 × 10^4^ cell/mL (individual channels and merge are shown). Scale bar = 500 μm. (**E**) Average ratio of hAELVi to NCI-H441 is shown across five biological replicates (n = 5) encompassing three fields of view ± SEM. The average ratio across the five replicates was 16.44:1, with the targeted ratio of 16:1 (grey line) displayed.

hAELVi and NCI-H441 are typically grown in huAEC and RPMI 1640 media, respectively, when seeding in monoculture. When seeded in co-culture at the theoretical seeding density, cells appeared to grow more rapidly after 72 h submerged and 24 h at the ALI in RPMI 1640 (NCI-H441) media compared to hAELVi media, or 50/50 media (Fig. 2A and B). Monoculture analysis reveals that hAELVi cells grow significantly more rapidly in NCI-H441 media than NCI-H441 cells, which were not impacted by medium type and, therefore, are likely attributable to the change observed in the co-culture cell numbers. This is potentially because the RPMI 1640 used as the NCI-H441 media is additionally supplemented with D-glucose, HEPES buffer, L-glutamine, sodium bicarbonate and sodium pyruvate. D-glucose, sodium pyruvate and L-glutamine may provide the metabolic inputs required to enhance cell proliferation and/or survival^21–23^. HEPES buffer and sodium bicarbonate on the other hand could possess enhanced buffering capacity, enhancing cell growth through reducing negative feedback mechanisms^24^. These considerations should be viewed bearing in mind that the hAELVi media formulation is proprietary information.

A mixture of the two media types resulted in a statistically non-significant increase in growth rate in the hAELVi cells, with the cell count laying between the cell counts for hAELVi cells grown in huAEC or RPMI 1640 medium, though this was not replicated in the co-cultured cells.

Medium type did not significantly impact viability in either monocultures or co-cultures. Viability remained approximately 80% or above throughout. Brookes et al. (2021) is the only study, to-date, to co-culture hAELVi and NCI-H441 cells, where they used each cell line seeded at equal densities with Advanced DMEM (Gibco) media. The growth of each cell line was not quantified independently; however, cells were found to grow to form a monolayer in this medium.

Based on these findings, it can be deduced that media choice does not impact cell viability; therefore, media choice to take forward falls onto the impact of growth and how this affects the targeted 13:1–16:1 ratio, as well as the barrier function of the cultures.

### Barrier integrity of submerged hAELVi/NCI-H441 Co-Culture at the air-liquid interface

The media type did not significantly impact membrane integrity in either monoculture or the co-culture (Fig. 2C). Although, basal media was found to leak through the cell culture insert membrane into the apical compartment of the insert during all repetitions when the co-culture was cultured in 50/50 and huAEC media (data not shown). This was not seen when co-cultures were cultured in NCI-H441 specific media.

The blue dextran assay revealed that membrane permeability was not significantly different in any culture regardless of media type. However, NCI-H441 cells grown in hAELVi media showed a non-significant increase in barrier function. When taken into co-culture, however, this effect is lost, attributable to the reduced number of NCI-H441 cells compared to hAELVi cells.

Co-cultures and hAELVi monocultures cultured at ALI were not possible to maintain, with media rapidly translocating from the basal compartment to the apical compartment. This phenomenon is not indicated in the blue dextran studies, perhaps indicating a one-way transport of solutes, or that the intercell junctions were not as tight as in the monoculture format.

Based on the above findings, a 50/50 media mix was chosen for future characterisation. This was chosen because the growth rate was not significantly altered, which did occur when hAELVi cells were cultured in RPMI 1640. Further, changes in the barrier function of hAELVi cells or co-cultures cultured in huAEC media were reduced. The 50/50 media resulted in growth, viability, and barrier function (*via* blue dextran) that were not significantly different compared to each individual cell type grown in appropriate medium.

### Co-culture seeding density optimisation

Given the inability to culture these cells at 2.5 × 10^5^ cell/mL in 50/50 media at the ALI due to the permeation of basal media into the apical compartment, it was considered that the 50/50 media mix may increase the time taken for a strong barrier formation. Whether due to the media type or the interaction between cell types in the co-culture. Therefore, increasing the initial seeding density was investigated to assess whether this could further increase barrier function, allowing a co-culture to be maintained at the ALI. Doubling the seeding density to 5 × 10^5^ cell/mL ensured enough cells were present to form a strong barrier, as shown in the blue dextran assay data (Fig. 2F).

Monocultures of either NCI-H441 ^17^ or hAELVi (Supplementary Figs. 2 and 3) have previously been characterised to form a confluent, tight monolayer at a 96-hour time point when seeded at 2.5 × 10^5^ cell/mL. When seeding at 5 × 10^5^ cell/mL, compared to 2.5 × 10^5^ cell/mL in the co-culture, cultures contained significantly more cells and formed a significantly tighter barrier (Fig. 2F) whilst maintaining similar percentage viability (85–90%). The experimental issue of ATI/ATII co-cultures not maintaining an ALI when seeded at a typical monoculture seeding density has arisen in the past during the development of a transformed type-1 (TT1) and NCI-H441 co-culture at anatomically relevant ratios. In this example, compared to respective monocultures, co-cultures also had to be seeded at twice the density to ensure that the ALI could be maintained^17^.

### Deducing seeding concentrations of hAELVi and NCI-H441 Co-cultures

Human surface coverage ratios of ATI: ATII are 13:1–16:1 ^8^. Although a theoretical seeding density had been calculated to obtain a 13:1–16:1 ratio at the time of the exposure based on the growth rate of each cell type in monoculture, reaching this ratio was not straightforward. Many factors likely contributed to this. The first is that each cell type likely grows at different rates. Although this was accounted for in the calculations based on the monoculture characterisation data, many factors can alter cell culture growth, such as temperature, pH, CO_2_ and O_2_ concentrations, and availability of fresh media^25^. Further, monocultures of hAELVi and NCI-H441 were characterised in huAEC and RPMI 1640, respectively, though the co-culture uses a 50/50 mix of the two. Given that different cell types require differing factors for growth, it is conceivable that altering the media would, therefore, change the growth rate^26^. Further, media supplements such as FBS (used in the RPMI 1640) have been shown to have reproducibility issues, which could contribute further to this^27–29^. Others have however shown that this issue can be mitigated through the use of FBS-free medium, replacing for non-animal-derived, chemically defined serum options^30,31^.

As described previously, the model was required to achieve a hAELVi: NCI-H441 surface coverage ratio of 13:1–16:1 ^8^. Initially, ATI and ATII-specific targets were identified and targeted using antibodies. Caveolin-1 is recognised as an ATI-specific marker^32^. 

**Table 1:** Seeding Densities investigated for use within the alveolar epithelial co-culture.

Cell Type	Cell Concentration Seeded (cell/mL)		
	Ratio 1	Ratio 2	Ratio 3
hAELVi	4.00 × 10^5^	4.25 × 10^5^	4.50 × 10^5^
NCI-H441	1.00 × 10^5^	7.50 × 10^5^	5.00 × 10^5^

However, when tested, caveolin-1 staining intensity appeared to be similar in hAELVi compared to NCI-H441 and, therefore, was unsuitable for use in deducing specific cell populations in co-culture (Supplemental Fig. 4). For this reason, the cells were instead pre-stained prior to seeding three differing ratios of hAELVi and NCI-H441 (Table 1) to assess which seeding density would closely resemble the 13:1–16:1 ratio at 96 hours (i.e., the point at which the model would be exposed for toxicological assessment).

Using CellTrackers to quantify the surface coverage of each cell type, seeding ratio 1, ratio 2 and ratio 3 had 4.8, 6.9 and 13.6 µm^2^ of hAELVi cell coverage, respectively, for every 1 µm^2^ of NCI-H441 cell coverage (Fig. 3B and C). Based on this, ratio 3 was taken forward for future work, which had seeding ratios of 4.5 × 10^5^ and 5 × 10^4^ cell/mL hAELVi and NCI-H441, respectively, which was found to give a surface coverage ratio of 13.6:1 (hAELVi: NCI-H441) (Fig. 3). This is close to the initially targeted ratio based on human anatomy data from Crapo et al. ^8^.

Further investigation of ratio 3 over 5 biological replicates (using 3 fields of view (FOVs) per replicate) found the average ratio of hAELVi: NCI-H441 was 16.44:1 ± 3.29 (Fig. 3D and E). This seeding ratio confirmed the initial findings and was therefore taken forward into future exposure studies.

Here, it has been shown that it is essential to track changes to the ratio of each cell type as the cultures grow, as the cell ratio at the time of exposure will likely not match the ratio of seeded cells. This area is not always reported or explored when co-cultures are presented, or the relevance to human anatomy is not commented on. For instance, Brookes et al. ^15^ used equal seeding ratios of hAELVi and NCI-H441 but does not characterise whether this ratio changes over time in culture.

### Adherence efficiency of dTHP-1 cells to alveolar epithelial cells at the air-liquid Interface and quantification of dTHP-1 density in the triple cell Co-culture

There should be 1 dTHP-1 per 18 × 10^3^ µm^233^ on the apical surface of the alveolar model. During the seeding of this model, dTHP-1 cells were added to an epithelial co-culture, allowed to adhere for 2 h, and then taken to the ALI (by removing the apical media). It could not be assumed that during this 2-hour adherence period 100% of the dTHP-1 cells would adhere. Therefore, the number of the cells in the apical media (removed when taken to the ALI) was counted to assess how many dTHP-1 had adhered. This was compared to cultures that had been cultured for the 2 h in dTHP-1-free conditions to allow for confounding detached epithelial cells.

**Fig. 4:**
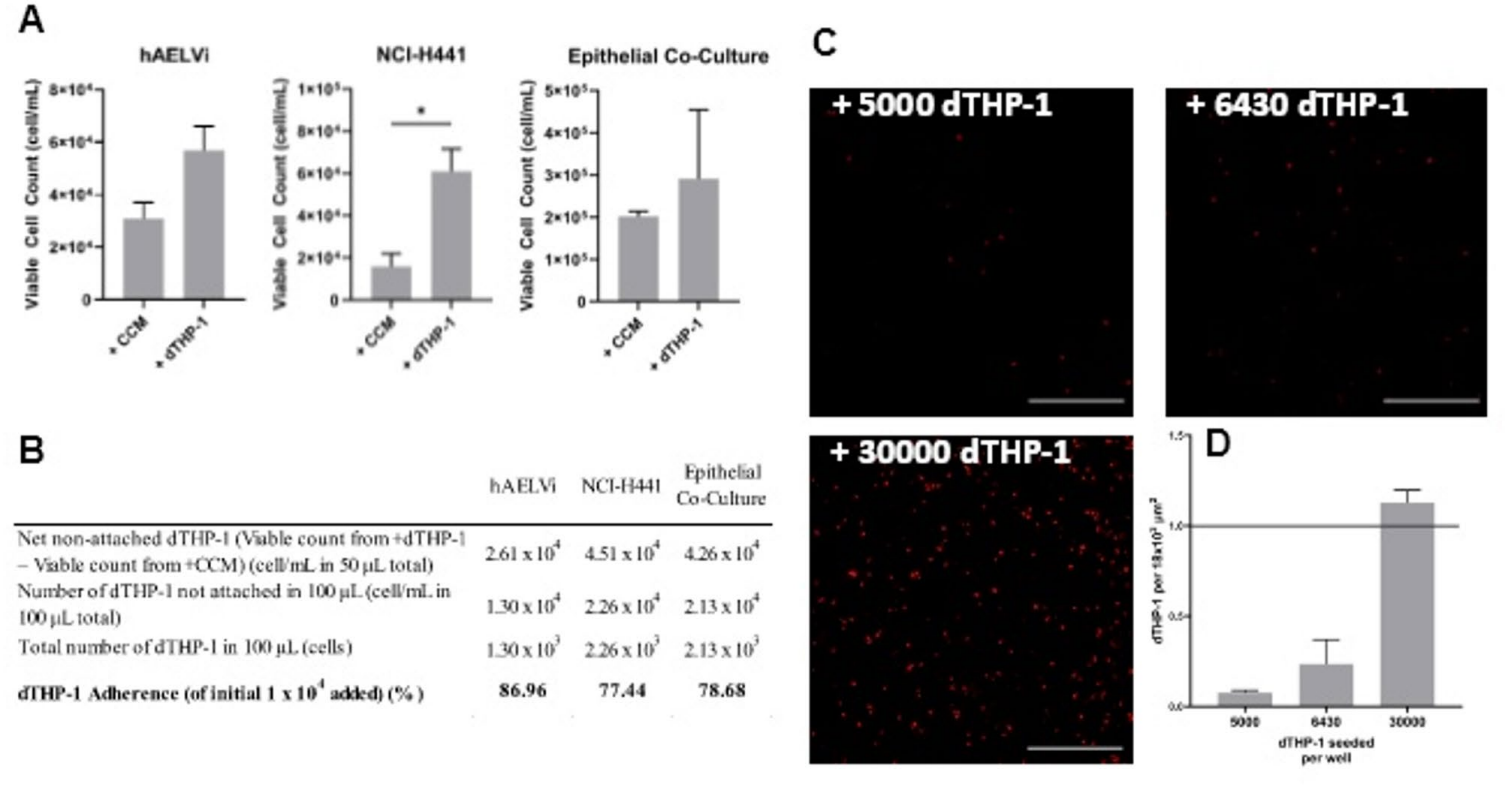
dTHP-1 cell adherence efficiency. (**A**) Viable cell counts using the LUNA II on media removed when cells were exposed to ALI two hours following the addition of dTHP-1 cells or cell-free media (CCM) to hAELVi, NCI-H441 or epithelial co-cultures. Bars show mean ± SEM across three biological replicates (n = 3). Significance was evaluated using unpaired t-test * = *p* ≤ 0.05. (B) displays the number of non-adhered dTHP-1 cells after accounting for the confounder of detaching epithelial cells. This is then used to calculate dTHP-1 adherence efficiency which advises the required seeding density of dTHP-1 cells. (**C**) CellTracker staining of dTHP-1 in the triple cell co-culture. Scale bars = 500 μm. (**D**) Number of dTHP-1 cells per 18 × 10^3^ µm^2^ shown as mean ± SEM (*n* = 2 for 5000 and 6430, *n* = 3 for 30000). The targeted density of dTHP-1 cells (1 cell per 18 × 10^3^ µm^2^) is shown.

When cultured on top of either hAELVi, NCI-H441 or epithelial co-cultures, there were always more viable cells counted in the discarded media after the addition of dTHP-1 cells (only reaching significance when cultured on top of NCI-H441 cells) (Fig. 4A). Viable cell counts enabled the percentage of adhered dTHP-1 to be calculated (Fig. 4B). Within this triple cell co-culture, macrophages were added on top of the hAELVi + NCI-H441 co-culture, which showed an adherence efficiency of approximately 79%, therefore a loss of approximately 21% of the dTHP-1 cells when taken to the ALI.

Given a requirement to have 1 dTHP-1 per 18 × 10^3^ µm^2 33^, it was therefore calculated that 6430 dTHP-1 cells were needed to be added per well to reach the targeted density whilst accounting for the loss of non-adhered cells. Using CellTrackers to pre-stain the dTHP-1 cells, it was found that actually 30,000 dTHP-1 cells needed to be seeded to achieve 1.13 dTHP-1 per 18 × 10^3^ µm^2^. Seeding with 5000 or 6430 dTHP-1 resulted in 0.06 and 0.24 dTHP-1 per 18 × 10^3^ µm^2^, respectively. Therefore, 30,000 dTHP-1 cells per well were instead used when seeding this model (Fig. 4C and D). The number of dTHP-1 required to be seeded was almost five times higher than expected, even considering non-adhered cells. This was interesting as when the dTHP-1 cells were added, they were checked for viability and cell suspensions were diluted based on the viable cell count. Using co-cultures of Calu-3 cells and dTHP-1, He et al.^34^ found that dTHP-1 numbers decreased over nine days with increased apoptotic bodies. This could suggest that a proportion of dTHP-1 cells may lose viability immediately upon seeding within the culture, perhaps explaining why a higher seeding density was required to reach the desired density 24 h later, though this study examined longer time durations than those used in the present work. The study also demonstrated that giving dTHP-1 cells 24 h to adhere to the epithelial cells before taking to the ALI resulted in increased formation of extensive multinucleated cell fusions compared to a 4 h adherence period. In this model, a 2 h adherence time was used, so the risk of this occurring is reduced here.

The co-culture model from He et al. utilised 1.0 × 10^4^ dTHP-1/cm^2^ (approx. 1 dTHP-1 per 1 × 10^4^ µm^2^), almost half as many macrophages per unit area than the model presented here, though this is a co-culture model of the conducting airways using macrophage histology data that is different from alveolar histology^35^. Kletting et al.^36^ seeded hAELVi and THP-1 cells at a ratio of 3:1 based on work by Stone et al.^33^; hAELVi were grown for 14 days before the addition of dTHP-1 cells which were suspended in 6 µL of media and incubated, without the removal of the additional apical media to form an ALI culture, or were added suspended in 500 µL media to form a submerged culture. It was found that there were fewer cells in the ALI culture, though the viability data for unexposed cultures was not shown. This, perhaps, again shows why more dTHP-1 cells than expected were required to be added to the co-culture.

### Assessment of dTHP-1 activation within the triple cell Co-culture

**Fig. 5:**
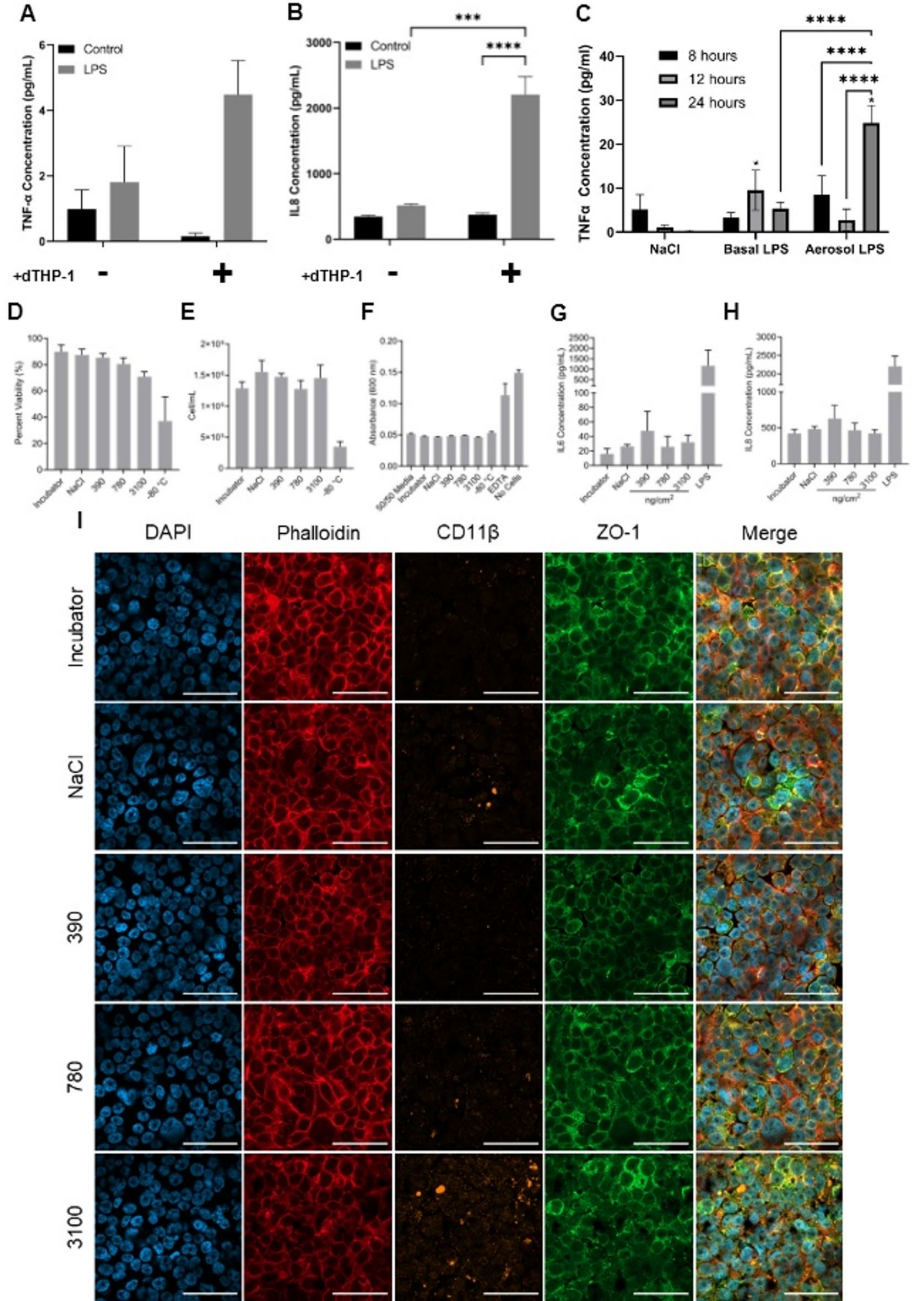
(Pro)-inflammatory response from the triple cell co-culture in response to TNF-α and Printex 90 carbon black. TNF-α (**A**) and IL-8 (**B**) release following lipopolysaccharide (LPS) stimulation of epithelial co-cultures or triple cell co-cultures (epithelial co-culture + dTHP-1 cells). (**C**) TNF-α release following LPS exposure either on the basal or apical (via aerosol) membranes. (**D**), total cell count (E), blue dextran (F), IL-6 concentration (**G**) and IL-8 concentration (**H**) of the triple cell co-culture exposed to CB using a VITROCELL Cloud at 390, 780 and 3100 ng/cm^2^. Bars represent the mean of three biological replicates (n = 3) with significance assessed using a one-way ANOVA utilising a Tukeys post hoc test. * *p* ≤ 0.05, ** *p* ≤ 0.01, *** *p* ≤ 0.001, **** *p* ≤ 0.0001. (I) Morphological examination of the triple culture following CB exposure, stained for nuceli, cytoskeleton, CD11β and ZO-1.

Once the correct number of dTHP-1 cells were confirmed within the triple cell co-culture, the models’ ability to react to a (pro-)inflammatory stimulus was assessed. For this, LPS (1 µg/mL basal exposure or 175 µg/mL nebulised using the VITROCELL Cloud) was used as a positive control to induce a (pro-)inflammatory response. When epithelial co-cultures or triple cell co-cultures were exposed to LPS, it was found that TNF-α levels increased in both epithelial cell co-cultures and the triple cell co-culture, though to a much greater extent in the latter. LPS also induced significant increases in IL-8 secretion in the triple-cell co-culture compared to epithelial cell co-cultures exposed to LPS and unexposed triple-cell co-cultures (Fig. 5A and B). Therefore, the ability of the triple cell co-culture to mount a larger (pro-)inflammatory response following LPS stimulus, compared to untreated triple cell co-cultures or epithelial co-cultures (lacking dTHP-1 cells) is shown here. This indicates that the dTHP-1 cells can maintain functionality for at least 48 h post-seeding in this model. Although the long-term survival and functionality of macrophages cultured at the ALI requires is the basis for future work.

It was further observed that aerosol exposure of LPS at the apical surface of the triple cell co-culture using a VITROCELL Cloud system increased TNF-α release compared to a basal exposure after 24 h (Fig. 5C). This is of interest as within human exposure scenarios inhaled inflammatory stimuli would contact the apical epithelial surface in the first instance. Given this augmentation of TNF-α release upon apical aerosol exposure, it could indicate a polarity of this cell model, which has previously been shown to be of critical importance to the initiation of airway epithelial cell (pro-)inflammatory response^37^.

He et al.^34^ similarly found that co-cultures of Calu-3 and dTHP-1 at the ALI produced significantly more apical TNF-α, IL-1β and IL-10 compared to monocultures of Calu-3 when exposed to aerosolised LPS. Given that the triple cell co-culture herein forms a tight monolayer, aerosol exposure is perhaps more relevant as basal LPS might not reach the dTHP-1 cells that are apical to the epithelial cells. Regardless, a (pro-)inflammatory response was shown. Here, IL-8 levels were increased significantly compared to TNF-α following LPS exposure, which was not significantly increased (Fig. 5A and B). It is possible that LPS is inducing a release of TNF-α from the dTHP-1 cells, which binds to the TNF-α receptor (TNFR) on epithelial cells^38^. This could then be sequestering the TNF-α protein, explaining the lower TNF-α concentrations detected. Downstream of TNF-α/TNFR signalling, IL-8 could be transcribed through NFκB mediated mechanisms, explaining the significantly higher levels of IL-8 release shown following LPS exposure. This has been tested experimentally in A549 cells and HTB54 cells where TNF-α exposure directly induces IL-8 release^39,40^. Following similar trends, co-cultures of A549 and dTHP-1 can react to gaseous stimulants, with co-cultures releasing more IL-8 than A549 monocultures when exposed to ozone^41^. Similarly, co-cultures were more sensitive than monocultures when exposed to hematite, quartz and silica particles, shown through augmented IL-6 and IL-8 release^42^. This further indicated the ability of cell-to-cell communication within an in vitro cultures, to modulate (pro)-inflammatory response^43^. Given that these models are reactive to both gases (i.e. ATI cells) and particles, it warrants using such models within this research to examine the (pro-)inflammatory effects of pollutant gases, PM or other inhalable engineered nanomaterials.

**Fig. 6:**
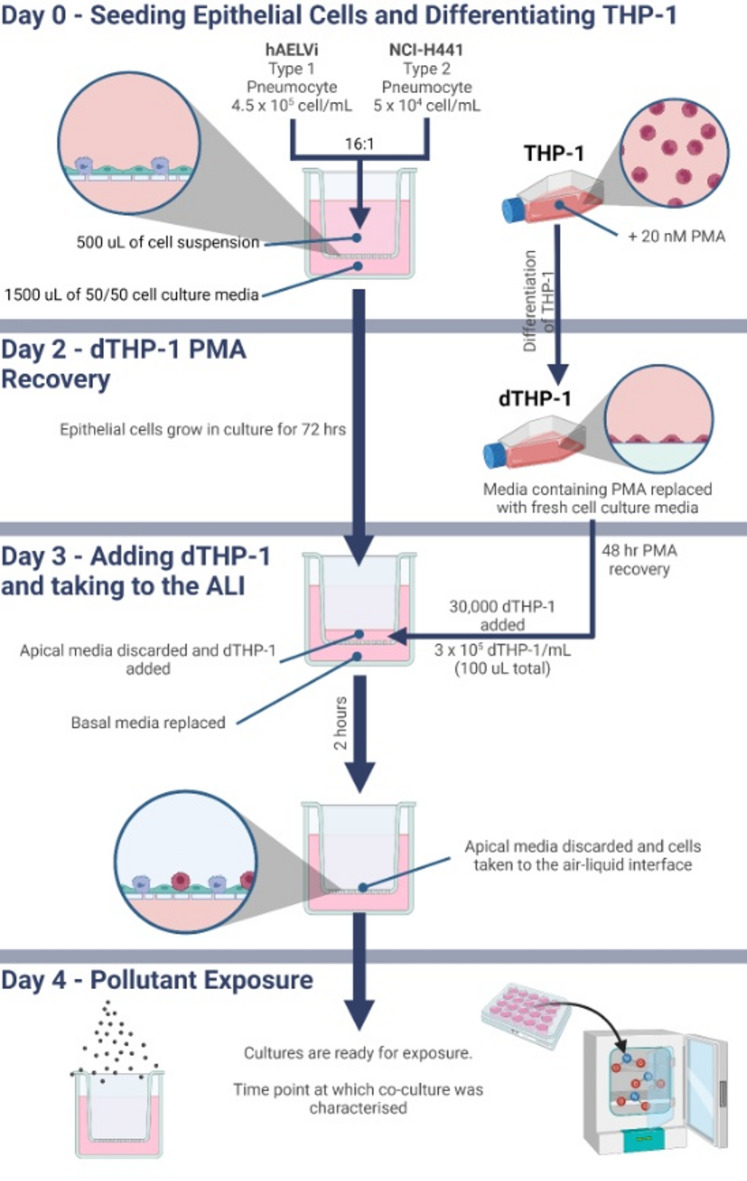
Schematic of the method for culturing the alveolar triple cell co-culture.

The approaches described, through cell density and media optimisation, surface coverage of each alveolar epithelial cell type, and dTHP-1 density and activation, an anatomically relevant alveolar triple cell co-culture has been developed to allow realistic exposures to inhaled xenobiotics or pharmaceuticals (Fig. 6).

To assess the response of the model to a foreign body exposure, CB was used as a model, surrogate particle of PM and delivered to the triple cell co-culture *via* aerosol exposure (VITROCELL Cloud). CB exposure caused a dose-dependent decrease in viability within the alveolar triple cell co-culture, approaching significance at the highest CB concentration (*p* = 0.061) (Fig. 5D and E). This could be related to CB having been shown to induce oxidative stress. Within A549 cells, CB can induce maximal ROS levels after 3 h (particle size dependent), which is also associated with the induction of cytotoxicity (Ryu et al., 2023). A high abundance of ROS within cells is associated with cell damage, namely, lipid peroxidation and damage to DNA and proteins. This could, in turn, lead to cytotoxicity.

Upon being challenged by CB, the membrane barrier integrity of the triple cell co-culture remained unchanged (Fig. 5F). It is possible that CB is unable to disrupt cellular barriers, though, it is also possible that hAELVi cells are highly resistant to barrier dysfunction, given that these cells are characterised to form tight junctions with a high TEER^32^, plus the tight barrier role these cells play within a physiological setting.

The triple cell co-culture was not able to mount a significant (pro)-inflammatory response to CB, although increased in both IL-6 and IL-8 were noted. Interestingly, a heightened release of both (pro)-inflammatory mediators was seen at the lowest exposure concentration (*p* > 0.05) (Fig. 5G and H). Initially, this lack of response was unexpected due to known pro-inflammatory potential of CB, however, given the complex interplay that is potentially occurring between cell types (and given the presence of macrophages in the presumed M1 phenotype), perhaps there are background inflammatory processes which are masking delicate particle induced (pro)-inflammatory responses. In addition, the deposited concentrations of CB used here are low when compared to other research. Previous work has shown neither A549 or differentiated THP-1 cells mounted an IL-8 response to the same grade of CB, even up to a concentration of 45.7 µg/cm^2^ (A549) and 22.8 µg/cm^2^ (THP-1)^44^. CB was however found to be inflammatory in murine models, contrasting with the effects observed in human cell lines^45^, perhaps a result of the differing anatomical and physiological differences mentioned previously. It is suggested that human-based new approach methodologies may be more relevant to toxicological testing than the use of traditional animal models^46^. Indeed, the work presented here aligns with frameworks that suggest holistic toxicological assessments using systems that reflect human biological systems could prove more relevant to human exposures^47^.

There were no apparent morphological changes following CB exposure in the triple cell co-culture. However, CD11β staining intensified with increased CB concentration (Fig. 5I).

Although these specific cells have not been used in a triple-cell co-culture model previously, other three or four cell models have been utilised within exposure studies. Costa et al. (2019), for example, investigated a triple culture containing NCI-H441 and dTHP-1 cells on the apical surface of cell culture inserts and then endothelial cells (HPMEC-ST1.6R) on the basal side of the membrane, utilising the model to assess translocation of nanoparticles across the air-blood barrier, finding that the presence of endothelial cells could increase the TEER of the model compared to a purely apical model. When stimulated with LPS, however, it showed that the dTHP-1 and endothelial cells were the primary cells involved in a (pro)-inflammatory response. In a similar model, Kasper et al. (2017) used A549 or NCI-H441 cells, dTHP-1 cells and endothelial cells (ISO-HAS-1). THP-1 cells were seeded in both an M1 and an M2 phenotype, finding that M1 may act as a model of inflammatory lung disease. Developing these co-culture models further, Klein et al. (2013) investigated a tetra-culture comprising A549, dTHP-1, endothelial (EA.hy 926) and mast (HMC-1) cells. In tetra-culture, these cells showed reduced ROS production compared to any of the monocultures when treated with an inducer of oxidative stress. When the model was exposed to silicon dioxide nanoparticles *via* aerosol exposure, uptake was only observed in the dTHP-1 cells. It was also noted that submerged conditions significantly increased IL-8 secretion of the model. The model has also shown sensitivity to both environmental (diesel emission particles) and engineered (silver nanoparticles nanomaterials^48,49^. All the data goes to show that the cells used within the model modulate the toxic response.

## Conclusion

A triple cell co-culture has been developed and highly characterised, ensuring that studies which have characterised human anatomy were used as the basis to inform the direction during the development of the herein presented co-culture.

The triple cell co-culture comprises three cell types: ATI, ATII, and alveolar macrophage model cells (hAELVi, NCI-H441 and dTHP-1, respectively). Understanding the exact cellular constituents at the time of exposure, as opposed to the time of seeding, is vital in understanding cell-specific effects. Though not characterised to the same extent, different combinations of cells within the triple cell co-culture can be seeded in co-culture going forward. This will allow investigation into further cell specific effects and interactions.

Regardless, the model has been shown to possess an anatomically relevant ratio of ATI: ATII and dTHP-1 per unit area. The model possesses a functional tight barrier and can react to a (pro)-inflammatory stimulus. Although in this study, the model was utilised within carbon black exposure studies, this model could also be used when looking at a range of emerging inhalation exposure hazards, such as engineered nanomaterial or micro/nanoplastics, used when assessing occupational risk hazard, within multi-pollutant/co-exposure studies, within temporal exposure approaches, or used when looking at effects of respiratory pathogens (e.g., Covid-19) and inhaled therapeutics.

## Supplementary Information

Below is the link to the electronic supplementary material.

Supplementary Material 1

## Data Availability

The datasets used and/or analysed during the current study available from the corresponding author upon reasonable request.
