# Supplementary material for: A human relevant in vitro alveolar epithelial barrier model to assess inhaled pollutant hazard
Source: Sci Rep. 2025 Nov 14;15:39909. doi: 10.1038/s41598-025-23768-4 (PMC12618939; doi:10.1038/s41598-025-23768-4)
Supplement: Supplementary file 1 — Supplementary Material 1 [file 41598_2025_23768_MOESM1_ESM.docx]

**Supplementary Material for: A human relevant in vitro alveolar epithelial barrier model to assess inhaled pollutant hazard**

Joshua W. P. Bateman^1^, Kirsty Meldrum^1^, Sarah M. Mitchell^1^, Ulla Vogel^2^, Martin J. D. Clift^1^*

*^1^In Vitro* Toxicology Group, Swansea University Medical School, Swansea, United Kingdom

^2^The National Research Centre for the Working Environment, Lersø Parkalle 105, DK-2100 Copenhagen Ø, Denmark

*Correspondence to:

**Professor Martin J. D. Clift**

*In Vitro* Toxicology Group,

Institute of Life Science 1,

Swansea University Medical School,

Swansea,

Wales, UK,

SA2 8PP

Email Address: m.j.d.clift@swansea.ac.uk

**A**

**B**

**C**

**F**

**E**

**D**

***Supplementary Figure 1 Characterisation of hAELVi cells in submerged conditions.*** *Total cell counts (A) viability (B), TEER (C) barrier integrity (D), IL6 release (E) and IL8 release (F) of hAELVi cells every 24 hrs for a total 144 hrs growth time. hAELVi cells were grown under sunder submerged conditions. Basal media was replenished at the 72-hr time point. Data points show the mean of each time point ± SEM over three biological replicated. Where error bars cannot be seen, this is due to the SEM bars being smaller than the data icon. Light microscopy images of hAELVi cells grown in a T75 using 4x, 10x, 20x and 40x lenses on an Invitrogen Evos XL Core. White arrows indicate cell narrow projections.*


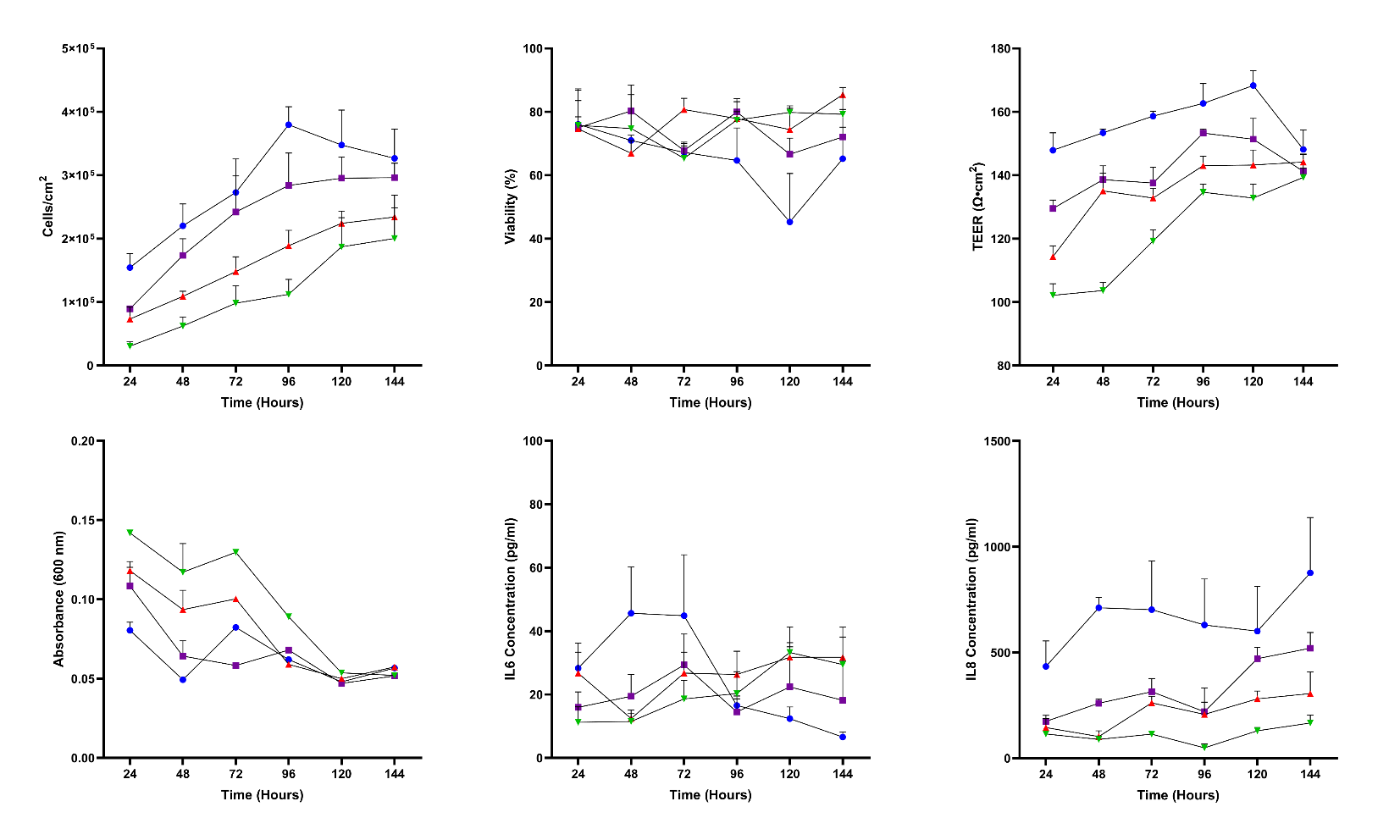

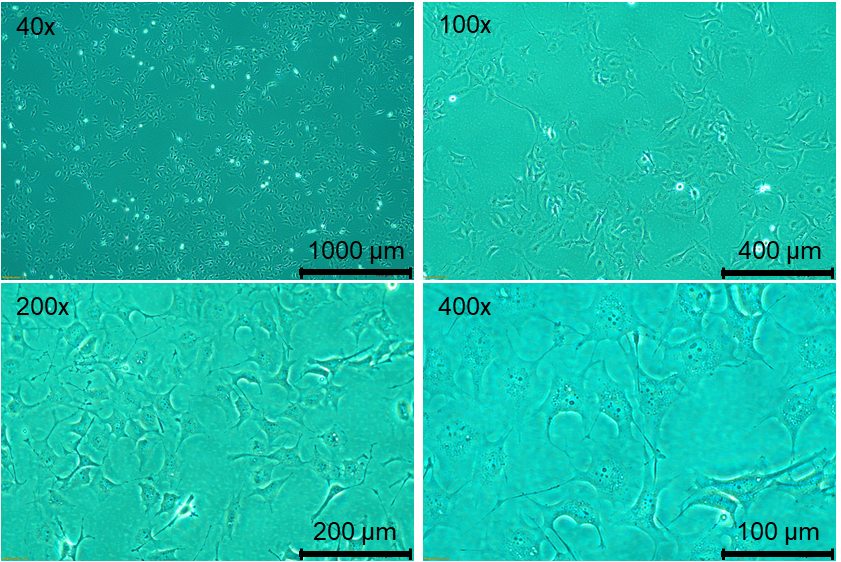

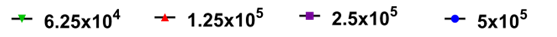


**Cell concentration (cell/mL):**


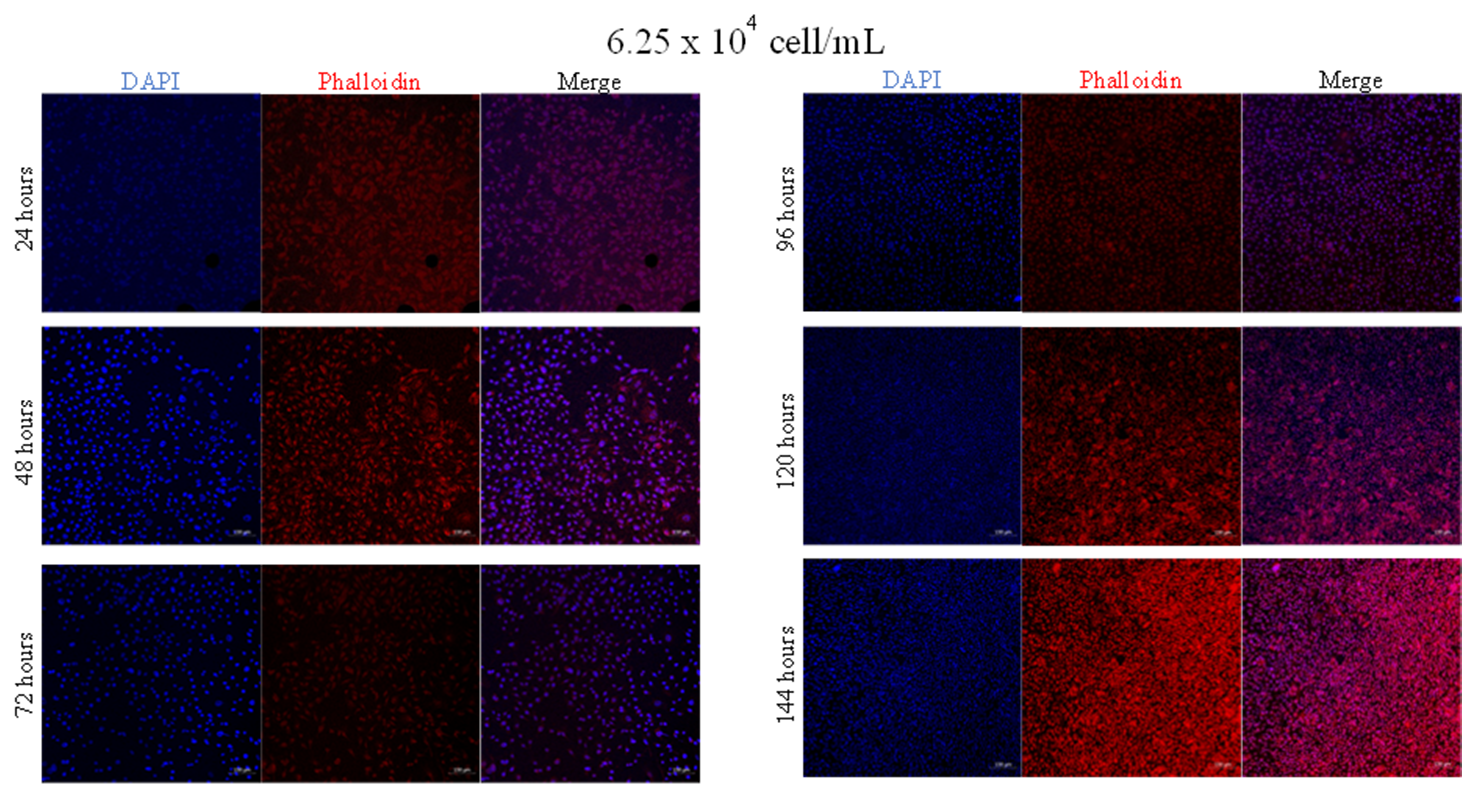

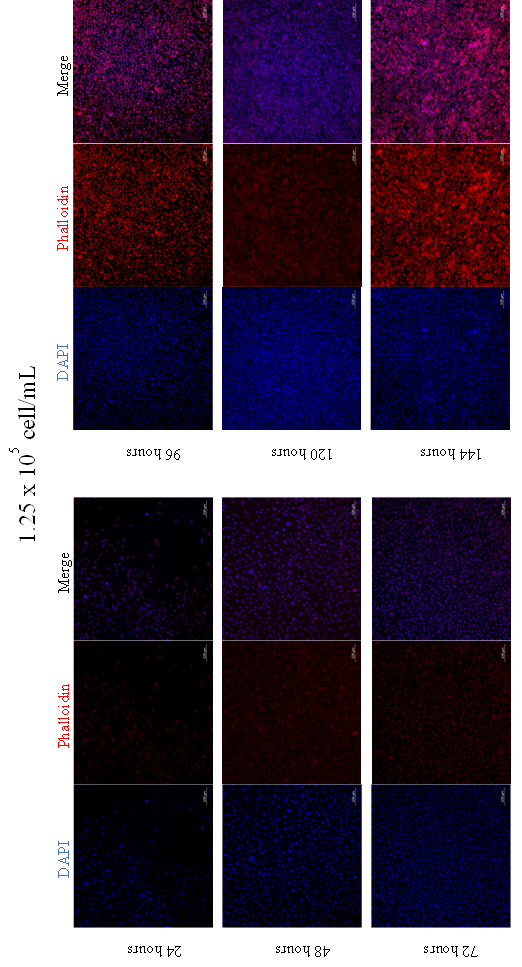

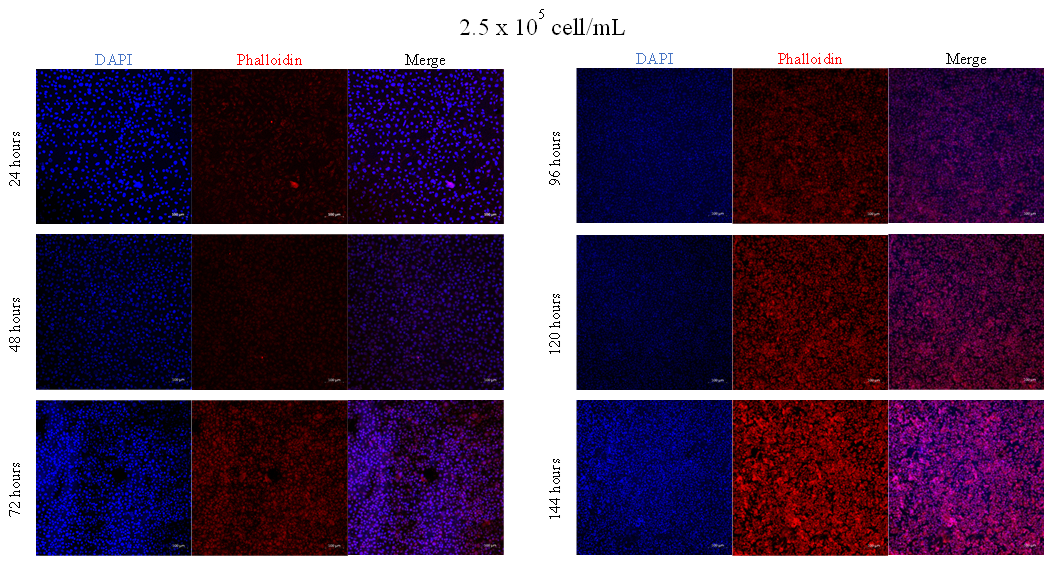

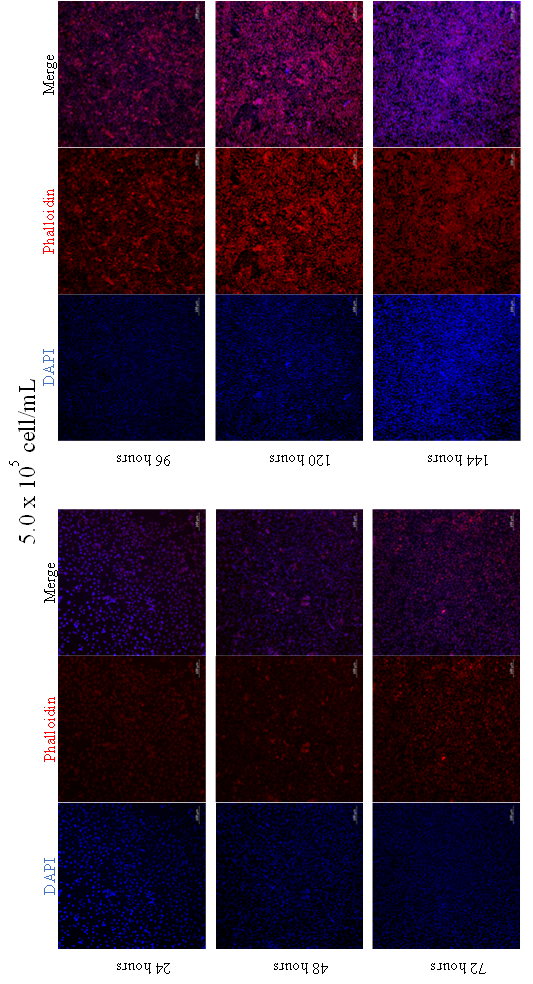


***Supplementary Figure 2 (and Previous pages) LSM images of submerged hAELVi cells were captured using 20x objective lens at 24-hour intervals.*** *hAELVi cells were stained using DAPI (nuclei) and phalloidin F-actin).*


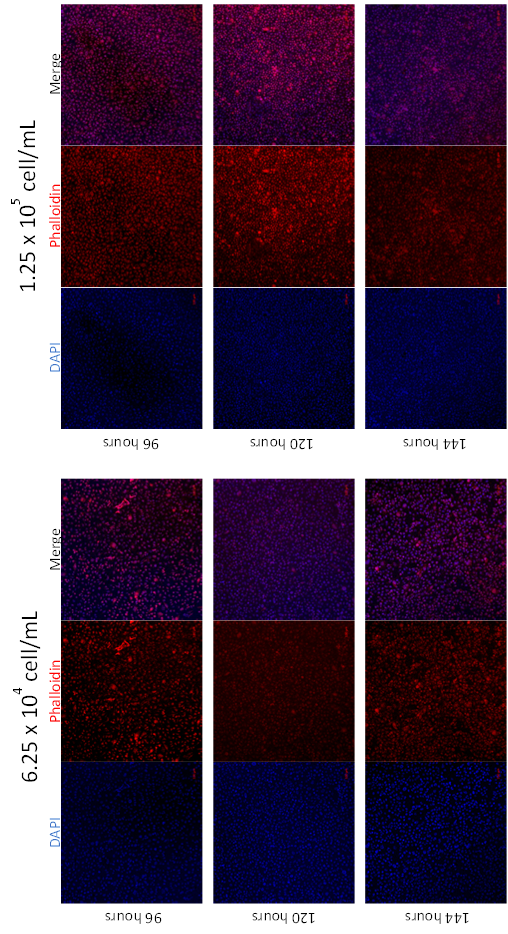

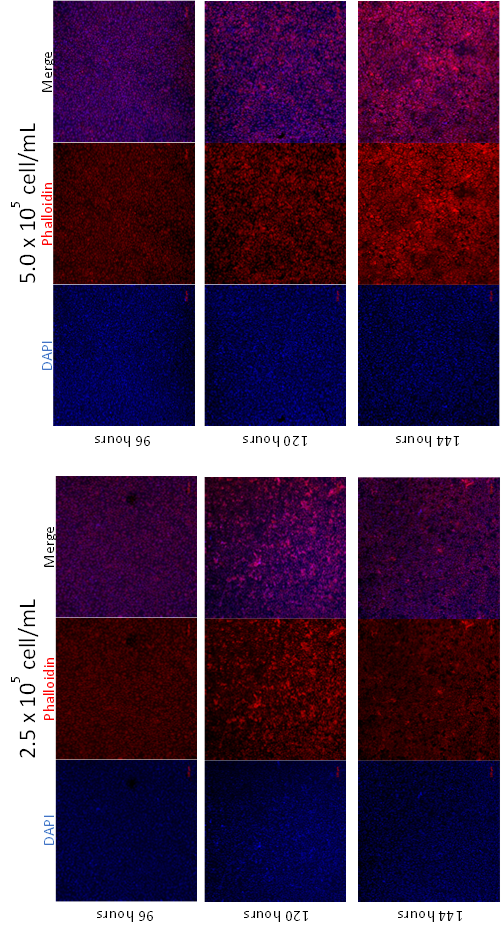


***Supplementary Figure 3 (and previous page) LSM images of hAELVi cells at the ALI were captured using a 20x objective lens at 24-hour intervals. hAELVi cells were stained using DAPI (nuclei) and phalloidin (F-actin).***


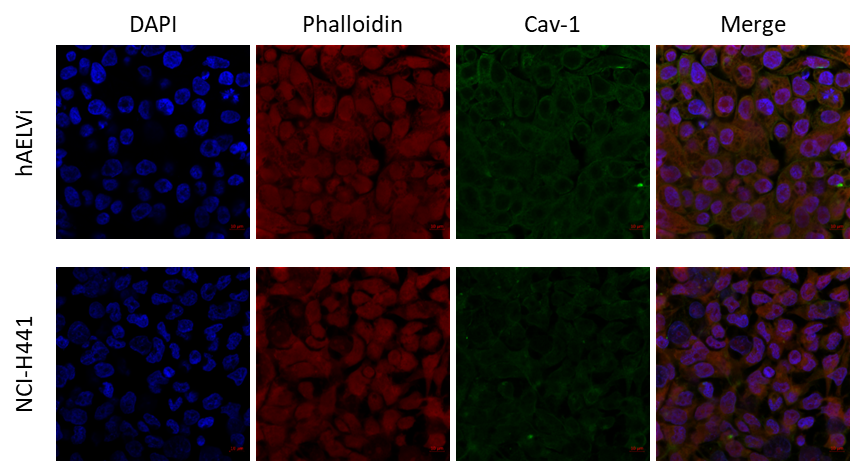


NCI-H441

hAELVi

DAPI

Phalloidin

Cav-1

Merge

***Supplementary Figure 4 LSM images of hAELVI or NCI-H441 monocultures using x63 objective lens to assess suitability for Cav-1 use in identification of hAELVi in co-culture. Scale bar = 100 μm.***
